# Supplementary material for: Intraocular Pressure Changes during Hemodiafiltration with Two different Concentrations of Sodium in the Dialysate
Source: Biology (Basel). 2021 Dec 23;11(1):12. doi: 10.3390/biology11010012 (PMC8773306; doi:10.3390/biology11010012)
Supplement: Supplementary file 1 [file biology-11-00012-s001.zip › Supplementary Material_R1.pdf]

Supplementary Material

Intraocular pressure changes during hemodiafiltration with two different concentrations of sodium in the dialysate

Based on the difference in IOP within the first hour, mean IOP in the fixed sodium profile group was 19.6 ±5.6 mmHg and individualized profile 17.8 ±3 mmHg, we used G*power software v. 3.1.9.7 to calculate the achieved statistical power of a t test with matched pairs. We computed an effect size of 0.56, considering an α error probability of 0.05. With this pilot study, we estimate that the required sample size to achieve a statistical power of 0.85, given this effect size, is 31 subjects (Figure S1).

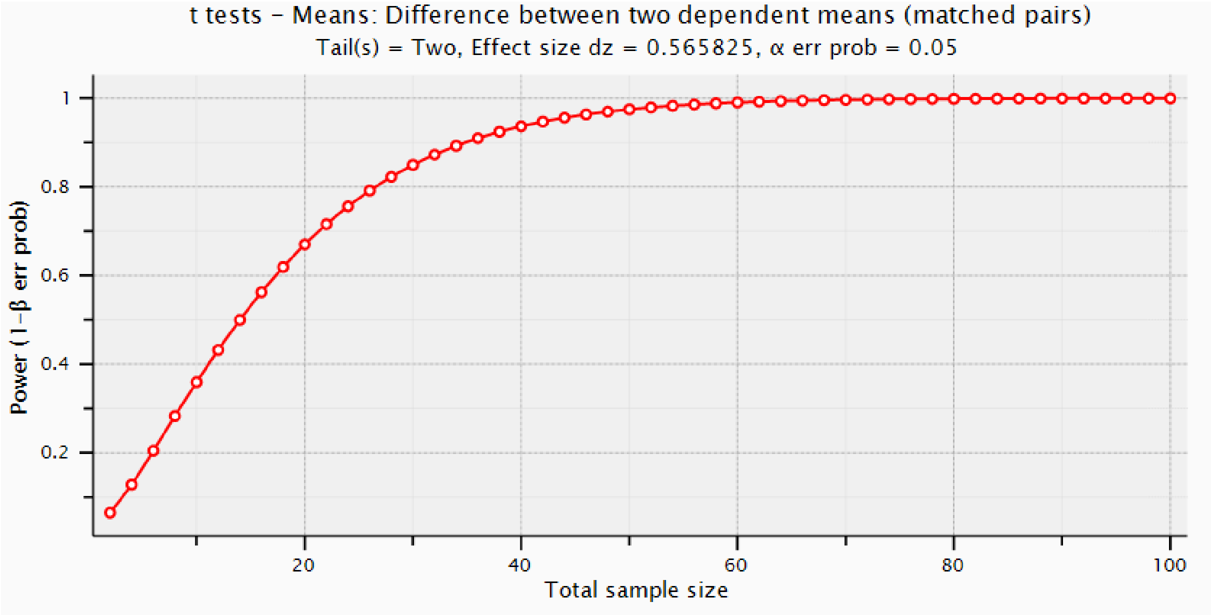

Figure S1. Achieved statistical power calculation.
